# Recurrent Intrahepatic Cholangiocarcinoma – Review

**DOI:** 10.3389/fonc.2021.776863

**Published:** 2021-10-21

**Authors:** Yuki Bekki, Dagny Von Ahrens, Hideo Takahashi, Myron Schwartz, Ganesh Gunasekaran

**Affiliations:** ^1^ Division of Liver Surgery, Recanati/Miller Transplantation Institute, The Icahn School of Medicine at Mount Sinai, New York, NY, United States; ^2^ Department of Surgery, Mount Sinai South Nassau, Oceanside, NY, United States

**Keywords:** intrahepatic cholangiocarcinoma, recurrence, management, risk factors for recurrence, re-resection of the liver

## Abstract

Intrahepatic cholangiocarcinoma (ICC) is the second-most common primary liver malignancy after hepatocellular carcinoma. While surgical resection with negative margin is the only curative treatment, ICC has very high rate of recurrence, up to 60-70% after curative resection. We reviewed the current data available on risk factors for ICC recurrence, recurrence pattern (location and timing), treatment options, and future directions. The risk factors for recurrence include elevated preoperative CA19-9, presence of liver cirrhosis, nodal metastasis, positive margins, and vascular invasion. Understanding different recurrence patterns, timing course, and risk factors for early recurrence is important to tailor postoperative surveillance and select treatment strategies including systemic or locoregional therapy. Re-resection can be considered for a selected patient population at experienced centers, and can yield long-term survival. ICC remains a dismal disease given the high likelihood of recurrence. Advances in our understanding of the genomic landscape of ICC are beginning to identify targetable alterations in ICC in subsets of patients that allow for personalized treatment.

## Introduction

Intrahepatic cholangiocarcinoma (ICC) is the second-most common primary liver malignancy, comprising of 5-10% of all primary liver cancers (1). Likely due to increasing use of cross-sectional imaging, its incidence has been increasing in the US and worldwide in the past several decades (2–5). Despite advance in systemic treatment (6, 7), surgical resection with negative margins is the only curative treatment for ICC (8–13). However, even with successful resection combined with adjuvant systemic chemotherapy, 5-year survival has ranged between 25-43% (8, 14–17) due to the high rate of recurrence. While the median survival after recurrence is approximately 12 months (14, 16), there is increasing evidence that aggressive multimodality treatment including re-resection may be prolong survival in selected patient populations (15, 16, 18).

Given the high recurrence rate, we aim to summarize the risk factors for recurrence, recurrence patterns, treatment options, and future directions in recurrent ICC management in this review.

## Risk Factors for Recurrence

Due to the heterogeneity of patients and tumor characteristics, management of ICC has to be tailored to the individual patient, including, for example, decisions about whether to employ adjuvant and/or neoadjuvant therapy (19, 20). Risk factors for recurrence in ICC have been extensively reported in the literature and include patient, histological, and treatment factors (21–24). The presence of underlying liver disease such as primary sclerosing cholangitis (PSC), viral hepatitis, and cirrhosis (21, 23) is a significant risk factor for both initial ICC incidence (25–27), and for increased recurrence after resection. Additionally, the presence of underlying liver disease can limit the ability to perform major resection which is often necessary in ICC to achieve oncologically optimal results (18). Elevated pretreatment carbohydrate antigen 19-9 is a marker of tumor aggressiveness and one of major risk factors for recurrence (28, 29).

Tumor-related risk factors include both gross characteristics like tumor size and number of lesions that are identifiable on imaging, and surgical margin status (30–33), vascular invasion (24, 29, 33) and regional nodal metastases (17, 24, 28, 29, 34) which are only identified histologically after surgery. Several nomograms have been reported to enable estimation of risk of recurrence based on tumor and patient risk factors (24, 29, 34).

Although recurrence risk is dependent on the treatment strategy, there are some controversies in this area.

### Routine Lymphadenectomy

While nodal metastasis is a major risk factor for recurrence, the role of routine lymphadenectomy remains controversial in ICC management. The American Joint Committee on Cancer (AJCC) recommends a lymphadenectomy with a minimum retrieval of 6 lymph nodes for ICC (35), since microscopic nodal metastases have been demonstrated in more than 40% of patients (17). However, given the complex pattern of lymphatic flow from the liver, complete regional lymphadenectomy is challenging (36). In a meta-analysis performed by Zhou and colleagues, lymphadenectomy during resection of ICC did not alter patient survival (37). In a review of data from the Surveillance, Epidemiology, and End Results (SEER) database (38), Kizy et al. found similar median survival for patients with nodal metastasis treated with surgical resection or with chemotherapy alone.

On the other hand, Altman and colleagues reported a positive impact of lymphadenectomy in another SEER database study. While systemic chemotherapy was associated with improved survival after resection in patients with nodal metastasis, patients who did not undergo lymphadenectomy were significantly less-likely to receive adjuvant chemotherapy (39). An international multi-institutional study found that patients with nodal metastasis who had ≥ three lymph nodes resected had an improved survival compared with patients with fewer than three nodes removed, suggesting a therapeutic effect of lymphadenectomy; the number of lymph nodes resected did not correlate with outcome in patients without nodal metastasis (40). Given the rather low sensitivity of preoperative cross-sectional imaging to diagnose lymph node metastasis, routine lymphadenectomy has been advocated for staging as well as possible therapeutic effect (41). Despite the AJCC recommendation, the performance and extent of lymphadenectomy during resection of ICC remain a topic of debate.

### Minimally Invasive Liver Resection

A recent retrospective study from a single institution used propensity score matching to demonstrate improved intraoperative and short-term outcomes, including number of nodes retrieved and depth of resection margin, with laparoscopic compared to open resection for ICC (42). Median disease-free survival (DFS) and overall survival (OS) were similar between the groups (DFS; 28 *vs*. 32 months, OS; 44 *vs*. 41 months). A recent meta-analysis of eight retrospective cohort studies confirmed the benefit of laparoscopic resection, showing a comparable number of nodes retrieved, a lower rate of positive margins, and improved DFS compared to open resetion (43).

On the other hand, a study based on the National Cancer Database (NCDB) found that patients who underwent laparoscopic resection more commonly had inadequate nodal sampling (laparoscopic 61% *vs*. open 39%; p<0.001) (44). The majority of studies advocating a minimally-invasive approach are single institution, retrospective studies and are thus highly heterogeneous and prone to selection bias (45, 46). At this point we can safely conclude that a minimallyinvasive approach is safe and feasible for selected patient populations at experienced centers.

### Routine Systemic Chemotherapy

The use of adjuvant chemotherapy after resection of ICC has long been controversial, as results of trials have been mixed (47). The BILCAP trial, reported in 2019, demonstrated improved survival with adjuvant oral capecitabine therapy in a protocol-specified sensitivity analysis for a population comprising patients with a mix of intra- and extrahepatic cholangiocarcinoma and gallbladder cancer, but failed to meet its primary endpoint of overall survival in the intention-to-treat analysis (7). After gemcitabine plus cisplatin was established as first-line treatment for advanced biliary tract cancer based on the ABC-02 trial (6), gemcitabine plus oxaliplatin (GEMOX) was studied in the adjuvant setting in the PRODIGE 12-ACCORD 18 trial, and the regimen failed to demonstrate benefit after resection of biliary tract cancer (48).

Although the routine use of adjuvant chemotherapy remains controversial, it is commonly employed in patients where pathology reveals high-risk features including positive lymph nodes and/or positive margins (18, 24, 29, 34, 49, 50).

While there have been no randomized trials of neoadjuvant systemic therapy in ICC, several retrospective studies have been reported, especially in the setting of initially unresectable tumors. A multicenter retrospective analysis demonstrated comparable OS and DFS between patients who did or did not receive neoadjuvant chemotherapy despite the fact that the patients who received neoadjuvant therapy initially had more advanced disease (20). Two retrospective studies document the potential for neoadjuvant chemotherapy to downstage initially unresectable tumors to where resection becomes feasible (51, 52). Future studies of neoadjuvant therapy in ICC will be helpful, though conducting prospective trials in resectable ICC has been challenging due to the low incidence and the heterogeneity of the disease.

## Recurrence Pattern

The high recurrence risk and poor prognosis of ICC is in large part the result of the disease only being discovered when it is relatively advanced locally; tumors are commonly large, and achieving complete resection is often technically challenging. Recurrence of ICC after curative surgical resection can occur at the resection margin, an intrahepatic site away from the margin, and/or extrahepatic organs; each manifestation has unique biology and patterns of progression. Furthermore, the timing of recurrence is also variable (53). Understanding different recurrence patterns, timing course and risk factors for early recurrence is important to tailor postoperative surveillance and to select treatment strategies including adjuvant therapy.

### Recurrence Location/Organ

A multi-institutional study of 920 patients with ICC found that 607 (66.0%) patients developed recurrence following curative resection. One hundred forty five patients (23.9%) recurred at the resection margin, 178 (29.3%) recurred intrahepatically away from the margin, 90 (14.8%) had extrahepatic-only recurrence, and 194 (32.0%) had both intra-and extrahepatic recurrence. Major extrahepatic recurrence sites include lungs, lymph nodes, peritoneum, bone, and adrenal. The different recurrence patterns had different time courses: intrahepatic margin recurrence and extrahepatic-only recurrence were commonly observed within 6 months, while intrahepatic recurrence away from the margin occurred gradually within 2 years (54).

### Recurrence Timing

The majority of ICC recurrence appears within two years of resection, and this is commonly defined as early recurrence (22, 23). Studies have demonstrated that recurrence patterns, risk factors, and outcomes differ significantly between patients with early *vs*. late recurrence. Not surprisingly, early recurrence is associated with worse prognosis (23). Tsilimigras et al. defined very early recurrence (VER) as recurrence within 6 months from initial resection based on distinct clinical features and more aggressive behavior noted in this group (21). Approximately one-quarter of patients with ICC in their series had VER, and their survival was dismal compared to those without VER (5-year OS 8.9% *vs*. 49.8%; p<0.001).

## Treatment of Recurrence

Although management of recurrent ICC is challenging and systemic therapy remains the cornerstone similar to patients who present primarily with advanced disease, several studies have reported benefit of incorporating aggressive locoregional treatment of recurrent disease compared to systemic therapy alone (15, 53).

### Re-Resection

The majority of ICC recurs in the liver, and re-resection in selected patients is associated with long-term survival (14, 22, 23, 55–57). A multi-institutional study of 400 patients with ICC recurrence demonstrated that those who underwent re-resection had a median survival of 26.1 months, compared to 9.6 months for nonsurgical locoregional treatment and 16.8 months for systemic chemotherapy (55). Another recent multi-institutional study of 113 patients who underwent re-resection for recurrent ICC demonstrated median survival of 65.2 months (58). While 156 patients who underwent repeated exploration for recurrent ICC were included in their study, 43 patients (27.6%) did not undergo re-resection.

Repeat liver resection for recurrent ICC is often challenging since initial ICC resections are commonly major resections, often with concomitant vascular/biliary resection and reconstruction, and with lymphadenectomy around the hepatoduodenal ligament (59). Patients selected for re-resection, in addition to a technically favorable situation, typically have had a long disease-free interval (often greater than two years), less-advanced initial stage, negative lymph nodes, and no extrahepatic disease (59, 60). There have been many single institution studies from around the world that have reported survival benefit of re-resection, and without question there are long-term disease-free survivors. However, the obvious selection bias inherent in operative candidates makes valid statistical comparison of re-resection with other treatment modalities impossible (14, 16, 56, 59–62).

### Locoregional Treatment

The use of locoregional treatments including thermal ablation (15), stereotactic body radiation therapy (SBRT) (63, 64), transarterial chemoembolization (TACE) and intraarterial yttrium-90 radiotherapy (16, 65), has been reported with varying degrees of success (66), and this remains an area of active investigation. **Table 1** summarizes the treatment modalities and corresponding outcomes for recurrent ICC (14, 55, 56, 58, 61, 63, 67–73). Zhang et al. reported comparable outcomes between thermal ablation group and re-resection group for recurrent ICC (median OS: 21.3 and 20.3 months, respectively). However, patients with recurrent tumor > 3cm demonstrated a higher OS rate in the re-resection group than those in the ablation group (67). Another single center retrospective study also identified a tumor size (> 2cm) as a risk factor for poor survival after thermal ablation for recurrent ICC (68).

**Table 1:** Treatment modality and survival after ICC recurrence.

Study	Treatment modality	No of patients	Size of tumor (cm)	Survival after recurrence (months)
Bartsch et al. (58)	re-resection	113	–	36.8
Si et al. (56)	re-resection	72	3	45.1
Zhang et al. (67)	re-resection	32	5	20.3
Yoh et al. (61)	re-resection	15	5	91.6
Zhang et al. (67)	ablation	77	–	21.3
Chu et al. (68)	ablation	40	1.5	26.6
Kim et al. (69)	ablation	20	1.5	27.4
Fu et al. (70)	ablation	12	3.2	30
Ge et al. (71)	TACE	183	6	26.9
Goerg et al. (72)	TACE	12	–	13.3*
Smart et al. (73)	radiation	66*	5.6	25*
Jung et al. (63)	radiation	30	–	13
Spolverato et al. (55)	chemotherapy	46	3	16.8
Park et al. (14)	chemotherapy	21	–	10

*Patients in both unresectable and recurrent ICC.

Intrahepatic cholangiocarcinoma, ICC; Transarterial chemoembolization, TACE.

TACE is another option with reasonable efficacy for unresectable recurrent ICC. A retrospective study of 275 patients with recurrent ICC included 183 patients who underwent TACE and 92 patients who underwent microwave ablation therapy. In their study, TACE provided longer survival after treatment than microwave coagulation therapy (median OS 26.9 *vs* 12.1 months). Interestingly, different prognostic factors for each treatment type were identified: the extent of tumor progression for TACE, and the etiologic subtype for microwave ablation therapy (71).

A meta-analysis of SBRT for unresectable or recurrent cholangiocarcinoma included 11 studies with 226 patients. The median OS was 13.6 months and 1-year local control rate was 78.6%, suggesting that SBRT was a feasible treatment option for those patients (64). These results are in line with the study by Jung et al. reporting the median OS of 13 months after SBRT for 30 patients with recurrent ICC (63). In order to apply higher dose of radiation towards tumors and reduce radiation related toxicity, proton radiation therapy have been introduced. Smart et al. demonstrated the efficacy of proton radiation therapy for 66 patients with unresectable or recurrent ICC with median OS of 25 months and 2-year local control of 84% (73). Even though radiation related toxicity can be a barrier to dose escalation, radiation therapy remains an effective local modality for recurrent ICC.

Although the level of evidence is limited due to the retrospective design and potential selection bias in these studies, locoregional treatment for recurrent ICC was associated with prolonged survival in patients with recurrent ICC (14–16, 22, 55, 59). With various locoregional treatment options available, comprehensive patient and tumor information is needed to stratify patients to select the treatment option including multimodal approach.

## Future Directions

With recent technological advances in Next Generation Sequencing (NGS), genomic profiling of tumors has become significantly easier and more affordable. As has been demonstrated in other cancer types (74, 75), molecular analysis of tumors can help clinicians to tailor the treatment for advanced or recurrent ICC (76, 77). The incidence of actionable mutations in patients with ICC ranges from 30-70%, with the most common being IDH1 and FGFR-2 (12, 78, 79). Similar to pancreatic cancer, targeting other genomic alterations such as DNA damage repair genes, HER2 amplification or activation, and NTRK gene fusions can improve survival compared to conventional systemic chemotherapy alone (74).

Immunotherapy has revolutionized cancer treatment and is currently being studied in ICC (80, 81). Identification of DNA mismatch repair deficiency on biopsy or surgical specimens is now routine, and as with other tumor types, these patients have a high rate of response to checkpoint inhibitors. While several biomarkers of response to immunotherapy have been identified, such as tumor mutation burden, presence of tumor-infiltrating lymphocytes, or programmed death-ligand 1 expression status (combined positive score) (82, 83), the response rate remains low (12, 78), and checkpoint inhibitors are generally given together with cytotoxic chemotherapy. As with most cancers, identifying biomarkers or genetic signatures of ICC that predict response to therapy is an area of intense research and will be integral to establishing an effective, personalized approach.

## Conclusions

ICC is the second most common primary liver malignancy with high risk of recurrence after curative resection. Risk factors for recurrence have been defined, and the majority of patients will have recurrent disease within 2 years of the initial resection. Prognosis after recurrence remains grim and treatment options beyond systemic treatment after recurrence are limited. While it can be technically challenging, repeat resection is a feasible and safe option for selected patients at experienced centers and can result in long-term survival. Other locoregional options such as thermal ablation, SBRT, TACE or intraarterial radioembolization increasingly being employed in conjunction with systemic therapy. Sequencing of tumor DNA is now routine in patients with ICC and can identify actionable mutations and genomic alterations that can help clinicians tailor treatment to manage this aggressive malignancy.

## Author Contributions

YB, DA, and HT drafted the manuscript MS and GG conceived the study and were in charge of overall direction and planning. All authors reviewed the results and approved the final version of the manuscript.

## Conflict of Interest

The authors declare that the research was conducted in the absence of any commercial or financial relationships that could be construed as a potential conflict of interest.

## Publisher’s Note

All claims expressed in this article are solely those of the authors and do not necessarily represent those of their affiliated organizations, or those of the publisher, the editors and the reviewers. Any product that may be evaluated in this article, or claim that may be made by its manufacturer, is not guaranteed or endorsed by the publisher.
